# Systematic literature review and meta‐analysis of concordance and accuracy of pretransfusion immunohematology routine tests

**DOI:** 10.1111/tme.70010

**Published:** 2025-08-25

**Authors:** Christopher Elliott, Stephanie Kelham, Liu Zhang, Stacy Grieve, Tommy Lan, Hoora Moradian, Cristina Coll‐Ortega, David Gómez‐Ulloa

**Affiliations:** ^1^ NHS Blood and Transplant, Newcastle Blood Centre Bristol UK; ^2^ Scientific Services LifeShare Blood Center Shreveport Louisiana USA; ^3^ Cytel, Inc. Cambridge MA USA; ^4^ Global HEOR & RWE, Grifols SA Sant Cugat Del Vallès Spain

**Keywords:** accuracy, antibody identification, antibody screening, blood typing, concordance, immunohematology, meta‐analysis, pretransfusion testing, sensitivity, specificity

## Abstract

**Objectives:**

This study aimed to assess the concordance and comparative accuracy of commercially available immunohematology (IH) tests for pretransfusion testing.

**Background:**

Pretransfusion tests are intended to ensure donor blood is matched with a compatible recipient. Automated testing has become the mainstay since the commercialisation of IH analysers, because of their reduced risk of human error and increased efficiency.

**Methods/Materials:**

A systematic literature review (SLR) was performed to identify studies evaluating the concordance and sensitivity/specificity of IH tests for ABO/RhD typing, antibody screening, or antibody identification. Pairwise meta‐analysis of concordance and sensitivity/specificity of IH tests was conducted when feasible.

**Results:**

The SLR identified 48 publications, of which 30 were included for meta‐analysis. ID/IH gel (Bio‐Rad), DG gel (Grifols), MTS/BioVue gel (Ortho Clinical Diagnostics), and Capture R (Immucor/Werfen) all had almost 100% pooled concordance with each other in ABO/RhD typing and antibody screening. Antibody identification results varied across studies, and pooled concordance rates were lower: 97.53% for DG gel versus ID/IH gel, 85.26% for ID/IH gel vs. MTS/BioVue gel, 85.54% for DG gel versus MTS/BioVue gel, and 71.19% for Capture R versus MTS/BioVue gel. For antibody screening, ID/IH gel, DG gel, and MTS/BioVue gel had pooled sensitivities of 94.23%, 96.31%, and 97.27%, respectively, with a pooled specificity of ~100% for all three tests.

**Conclusion:**

All tests had good concordance in ABO/RhD typing and antibody screening, and lower pooled concordance rates for antibody identification. For antibody screening, 95% pooled sensitivity and ~100% specificity were estimated for DG gel, MTS/BioVue gel, and ID/IH gel.

## 1 INTRODUCTION

Pretransfusion testing is a rigorous, multistep process to ensure donor blood is matched with a compatible recipient to allow for ample red blood cells to survive post transfusion.^1^, ^2^ The testing procedure is conducted with an aim towards minimising the risk of adverse reactions such as the development of haemolytic transfusion reactions, including ABO incompatible blood transfusions.^3^

Red blood cells have antigenic proteins and carbohydrates on their surfaces, and these epitopes are categorised into blood groups based on structural characteristics and similarities to a parent protein.^4^ Exposure to these antigens through events like pregnancy, transfusion, or transplantation can lead to the development of antibodies in individuals lacking specific epitopes. Subsequently, red cell alloantibodies may target and lyse transfused red cells possessing the corresponding antigen.^3^ Approximately 2% to 4% of a given population possess irregular or non‐ABO red cell alloantibodies, which are antibodies that are formed as part of an immune response rather than naturally occurring anti‐A or anti‐B^3^. Those can cause hemolytic disease in newborns or lead to the hemolysis of transfused red cells from the donor. These antibodies can be identified through pretransfusion testing, allowing for antigen‐negative units to be administered to the blood recipient.^3^ The pretransfusion testing process has three steps: ABO/RhD typing, antibody screening, and antibody identification (**Supplementary material** [Supplementary Figure 1]).^5^

Tube agglutination was first established as the standard method for pretransfusion testing.^6^, ^7^ In the past few decades, commercial kits of column or solid‐phase tests for pretransfusion purposes have progressively replaced conventional tube tests in most laboratories.^8^ With technology advancement, automated immunohematology (IH) test systems have increasingly become the mainstay for diagnostics.^9^ The conventional tube technique for blood group and cross‐matching is still considered a relatively easy‐to‐perform method and the gold standard; however, the method is limited by the variability of laboratory expertise and the inconsistency of the reporting of results.^10^, ^11^ The British Committee for Standards in Haematology guidelines recommend the use of fully automated systems, where possible, to reduce the introduction of human error and variable interpretation.^9^

Different IH analysers are available for pretransfusion testing. Although multiple studies have evaluated the concordance, sensitivity, and specificity of specific test systems,^6^, ^8^, ^12^, ^13^ there is a lack of synthesised evidence comparing their performance based on all available evidence.

The objectives of this study were to conduct a systematic literature review (SLR) to identify studies reporting the accuracy and concordance of different IH test systems in terms of ABO/RhD typing, antibody screening, or antibody identification; assess the feasibility of conducting concordance analysis and comparative accuracy analysis of commercially available IH tests for pretransfusion testing from studies identified through the SLR; and meta‐analyse the concordance between different IH tests and the sensitivity and specificity of each IH test in terms of ABO/RhD typing, antibody screening, and antibody identification.

## 2 MATERIALS AND METHODS

### 2.1 SLR

An SLR was conducted to identify studies reporting the accuracy and concordance of different IH test systems in terms of ABO/RhD typing, antibody screening, or antibody identification. The SLR was based on methodological guidance from the Preferred Reporting Items for Systematic Reviews and Meta‐Analyses (PRISMA) statement^14^ and Cochrane Handbook for Systematic Reviews of Interventions^15^ as well as from key health technology assessment bodies, such as the National Institute for Health and Care Excellence,^16^ Canada's Drug Agency,^17^, ^18^ and the Institute for Quality and Efficiency in Health Care.^19^

Database searches were conducted on April 19, 2023, in MEDLINE, Embase, and Evidence‐Based Medicine (e.g., Cochrane Central Register of Controlled Trials and Cochrane Database of Systematic Reviews) via the Ovid platform. Full texts and indexed abstracts published between 2006 and April 2023 were considered for inclusion. This time limit was applied to correspond with the introduction of automated analysers.^20^ The search strategies included a combination of free‐text and controlled vocabulary terms specific to each database (e.g., Emtree terms for Embase or Medical Subject Headings in MEDLINE).

The proceedings of the following key conferences were manually searched for any editions not yet indexed in Embase at the time of the search: International Society of Blood Transfusion, Association for the Advancement of Blood & Biotherapies, British Blood Transfusion Society, and the American Association of Clinical Chemistry.

The data sources for the SLR are provided in Supplementary material (Supplementary Table 1), and full search strategies can be found in Supplementary material (Supplementary Table 2).

The selection of studies was guided by the population, intervention, comparators, outcomes, and study design criteria (Supplementary material [Supplementary Table 3]). Any comparative study on human blood samples undergoing testing was considered for inclusion. The studies were required to compare at least two technologies (index and reference test must be clearly identified) for ABO/RhD typing, antibody screening, or antibody identification. Outcomes of interest included sensitivity, specificity, true positive/negative, false positive/negative, positive/negative predictive value, analysis of discordant results, concordance/discordance results, accuracy, and test time (e.g., turnaround, hands‐on time, maintenance).

Literature was screened at the title/abstract and full‐text levels by two independent reviewers, with conflicts resolved by a third, independent researcher. A data extraction template was developed in Microsoft Excel® (Redmond, WA, US) to capture study characteristics, IH test characteristics, and outcomes of interest. Included studies were check‐boxed for each type of outcome (ABO/RhD typing, antibody screening, antibody identification). Any necessary assumptions and/or calculations, including rationales and calculation details, were documented in the spreadsheet to note uncertainty around the data. Data were extracted by a single reviewer with verification by a second reviewer; any discrepancies were resolved via discussion. A higher‐level quality review was then performed by a senior researcher.

Handling of missing statistics and data conversion took place during the data extraction phase; each calculation underwent an extensive quality check.

Quality appraisal of the selected studies with full‐text availability was performed using the Quality Assessment of Diagnostic Accuracy Studies 2 tool. Four domains of diagnostic accuracy (patient selection, index test, reference standard, and flow and timing) were assessed for each study. Quality appraisal and risk of bias for all included studies were conducted by one reviewer, with the assessment undergoing full quality control by a second, senior reviewer. Quality assessment of conference abstracts was not performed due to the lack of available data.

### 2.2 Feasibility assessment

For pairwise meta‐analysis of concordance or meta‐analysis of sensitivity/specificity to be feasible, the same comparison or index test needed to be reported by at least two studies.

#### 2.2.1 IH tests of interest and outcomes of interest

Different IH analysers are available on the market for pretransfusion testing, primarily from four major manufacturers: Grifols, Bio‐Rad, Ortho Clinical Diagnostics, and Immucor. Therefore, four commercial systems plus tube testing (not as an index test) were considered IH tests of interest: DG Gel (Grifols, Barcelona, Spain), MTS/BioVue Gel (Ortho Clinical Diagnostics, Raritan, NJ, US), IH/ID Gel (Bio‐Rad, Hercules, CA, US), Capture R (Immucor/Werfen, Norcross, GA, US).

Technology names and analysers for each product differed across the included studies. Therefore, the IH tests were grouped by manufacturer to ensure an adequate number of studies for meta‐analysis. This approach was validated by experts in pretransfusion testing who have suggested that the differences among products by the same manufacturer were expected to be minimal due to the use of the same technology. Technologies and analysers as reported in the included studies are listed in Supplementary material (Supplementary Table 4).

Concordance and sensitivity/specificity were outcomes of interest in the current study since they were widely used in diagnostic test studies to assess the agreement between different tests and diagnostic accuracy, respectively.

In situations where two or more manufacturers' products were used as one of the comparators (e.g., Bio‐Rad/Ortho vs. Grifols) in a concordance pair, or two or more manufacturers' products were used as the index test (e.g., either Bio‐Rad or Ortho were used as the index test in a study but there were no separate data reported for each individual product) for sensitivity or specificity, the data were excluded from the current analysis.

Data were assessed for meta‐analysis based on concordance rates and sensitivity/specificity.

#### 2.2.2 Data for concordance analysis

Concordance data were extracted when a study evaluated the agreement between two IH tests from different manufacturers. Concordance data can be reported by concordance rate (i.e., number of agreed tests divided by the total number of tests) and by Kappa statistics.^21^

#### 2.2.3 Data for sensitivity and specificity analysis

Sensitivity (i.e., the proportion of true positives correctly identified by a test) and specificity (i.e., the proportion of true negatives correctly identified by a test) are usually assessed by comparing an index test to the gold standard. In general, sensitivity and specificity are negatively correlated across studies (i.e., the increase of one is expected to be associated with the decrease of the other); therefore, for the calculation of both sensitivity and specificity, the numbers of true positives, false positives, true negatives, and false negatives were required for meta‐analysis.

### 2.3 Meta‐analysis

#### 2.3.1 Concordance

For each concordance pair of IH tests, concordance rates were synthesised by meta‐analysis for proportions using R function “metaprop” within the “meta” package (version 6.5–0).^22^ The proportions were pooled using the generalised linear mixed model (GLMM). This approach fully accounts for the uncertainties within each study, does not require continuity correction, and performs well for analyses with small sample sizes and rare events.^23^ The GLMM method was used in this analysis considering the common existence of 100% or close to 100% concordance rate and the wide range of sample sizes across studies.

The count of concordance was directly modelled with binomial likelihoods, and logit link function was used to transform latent true proportions to a linear scale.^24^ Each concordance pair of IH tests was presented as a forest plot. The concordance rate with Clopper–Pearson confidence intervals (CI) was reported for each study.

Pooled concordance rates with 95% CIs were estimated using both a fixed‐effects model and a random‐effects model. High between‐study heterogeneity is common in meta‐analysis of diagnostic tests; random‐effects is the preferred approach since it is better suited than the fixed‐effects model to address this heterogeneity.^25^

The heterogeneity statistic *I* ^*2*^ was calculated to assess the heterogeneity among the studies^26^; *I* ^*2*^ around 25%, 50%, and 75% indicates low, medium, and high heterogeneity, respectively.^27^

The analysis result for each concordance pair is presented as a forest plot, with concordance rate and corresponding 95% CI. Both I^2^ and Tau^2^ are presented as heterogeneity statistics, as well as the *p*‐value.

Since the meta‐analysis of each concordance pair was conducted independently, the pooled concordant rates were not comparable across different pairs.

#### 2.3.2 Sensitivity and specificity

Meta‐analysis of sensitivity and specificity was conducted based on the recommendations by Cochrane Handbook for Systematic Reviews of Diagnostic Test Accuracy.^28^ A random‐effects bivariate binomial model was fitted as a GLMM using the “glmer” function from the R package “lme4” (version 1.1–35.1).^25^, ^29^, ^30^ This approach models logit‐transformed sensitivity and logit‐transformed specificity simultaneously, assuming that estimates from individual studies may vary while originating from a shared underlying distribution with an unstructured between‐study covariance matrix.

The assumption of bivariate normal distribution at logit scale for the random effects accounts for the potential correlation between sensitivity and specificity within studies.^31^

For each index IH test of interest, the sensitivities and specificities of the included studies were presented as a forest plot. The weight of each individual study was calculated using methods by Burke et al., 2018.^32^ From the bivariate binomial model, the pooled sensitivity and specificity with 95% CIs were reported.

All analyses were performed using R version 4.3.2 within the R Studio environment.

## 3 RESULTS

### 3.1 SLR

The database searches returned 1513 records, of which 211 were retained after title/abstract screening. Fifty‐three publications were included after full‐text screening, and two records were identified from congress searches. In total, 55 reports on 48 unique studies were identified by the SLR to be included for feasibility assessment. Data from the 48 included studies were used to assess the feasibility of conducting meta‐analysis for concordance and diagnostic accuracy of different IH tests. Thirty studies met the additional selection criteria, reporting on either concordance rate or sensitivity/specificity. Other reasons for exclusion included only turnaround time reported, mixed reference standard, and no two‐way comparison. The list of studies excluded after the application of additional selection criteria can be found in Supplementary material (Supplementary Table 5).

**FIGURE 1:**
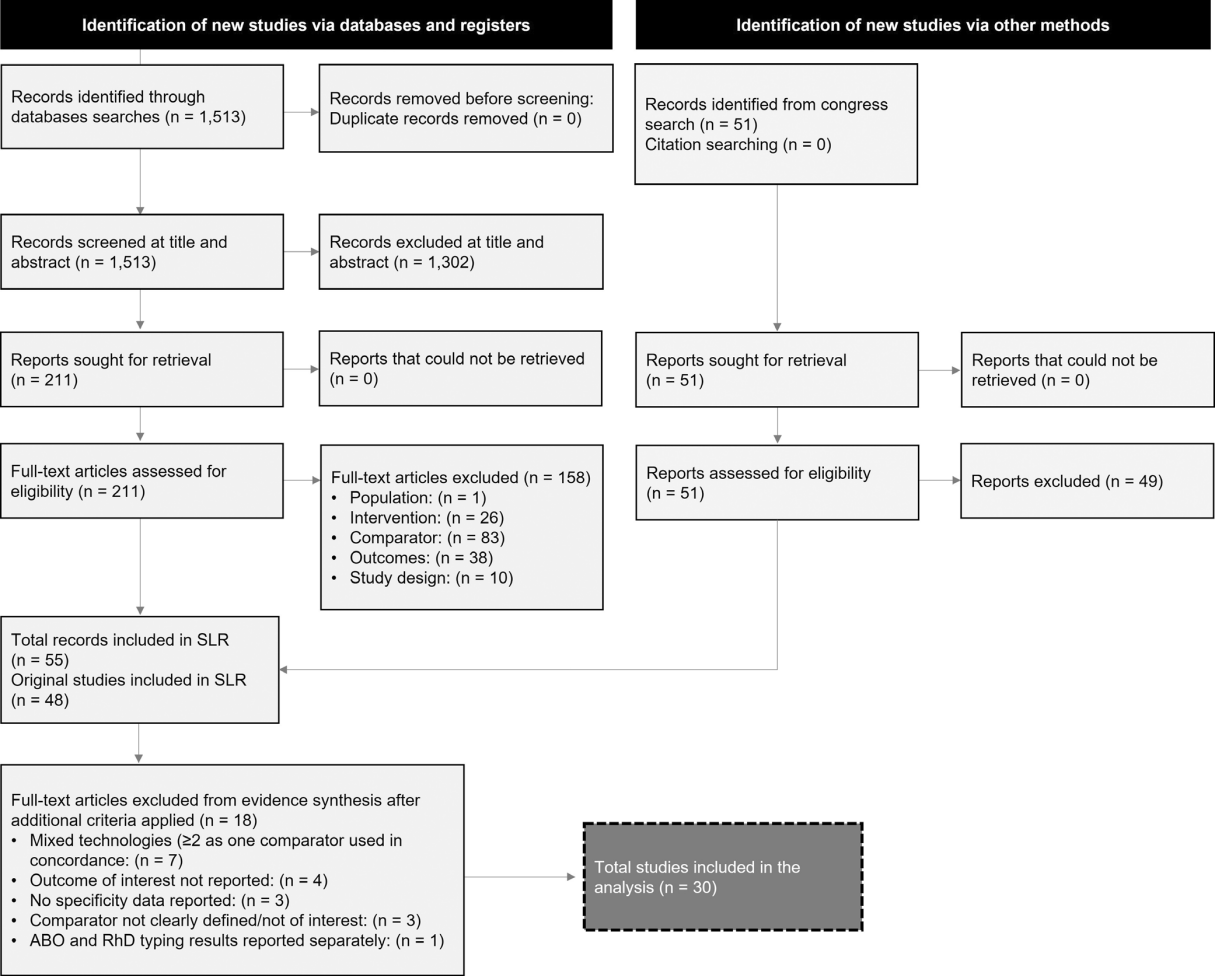
Preferred Reporting Items for Systematic Reviews and Meta‐Analyses diagram of systematic literature review.

The literature attrition is presented as a PRISMA diagram in Figure 1. An overview of the included studies is provided in Supplementary material (Supplementary Table 6).

The majority of studies (*n* = 24) were published as conference abstracts and the remainder (*n* = 6) were reported in full‐text publications; the lack of data from full‐text publications may introduce a certain risk of reporting bias. Nine studies were conducted in North America while the remainder were categorised as “rest of world” for the purposes of this study.

The overall risk of bias across the studies was deemed low. The full results of the quality appraisal for the diagnostic accuracy studies included in the meta‐analysis are summarised in Supplementary material (Supplementary Table 7).

### 3.2 Meta‐analysis

For concordance analysis, only two sets of test comparisons were reported from at least two studies (ID/IH gel vs. MTS/BioVue gel for ABO/RhD typing and DG gel vs. ID/IH gel for antibody screening)^6^, ^13^, ^33^, ^34^; therefore, due to the scarcity of data, further synthesis using Kappa statistics was not conducted.

Eligible concordance rate data are provided in Supplementary material (Supplementary Table 8 for ABO/RhD typing, Supplementary Table 9 for antibody screening, and Supplementary Table 10 for antibody identification). If a study reported IH tests from more than two manufacturers, concordance data for different combinations of tests were listed.

Overall, meta‐analysis was feasible for the concordance of ABO/RhD typing, antibody screening, and antibody identification.

Sensitivity and specificity data for ABO/RhD typing was reported by only two studies, both of which reported on ID gel (Supplementary material [Supplementary Table 11]); therefore, the data were not further synthesised.

Sensitivity and specificity of antibody screening were reported in nine studies (Supplementary material [Supplementary Table 12]).

In the studies that reported antibody identification, the majority of them focused on the samples that were already antibody screen positive, leading to only sensitivity data available; therefore, further data synthesis could not be conducted due to the lack of specificity data.

Meta‐analysis was feasible for the sensitivity/specificity of antibody screening, but there was a lack of common reference standards across studies (Supplementary material [Supplementary Table 13]), limiting their comparability. There is currently no gold standard for antibody screening; therefore, data synthesis was performed in the context of this limitation. The reference standard used in each study was considered to show the true value; therefore, this assumption was applied to enable determination of which of the discordant results between tests of different manufacturers were the true value. In some cases, a comparator was assumed as the reference standard.

#### 3.2.1 Concordance of ABO/RhD typing

**FIGURE 2:**
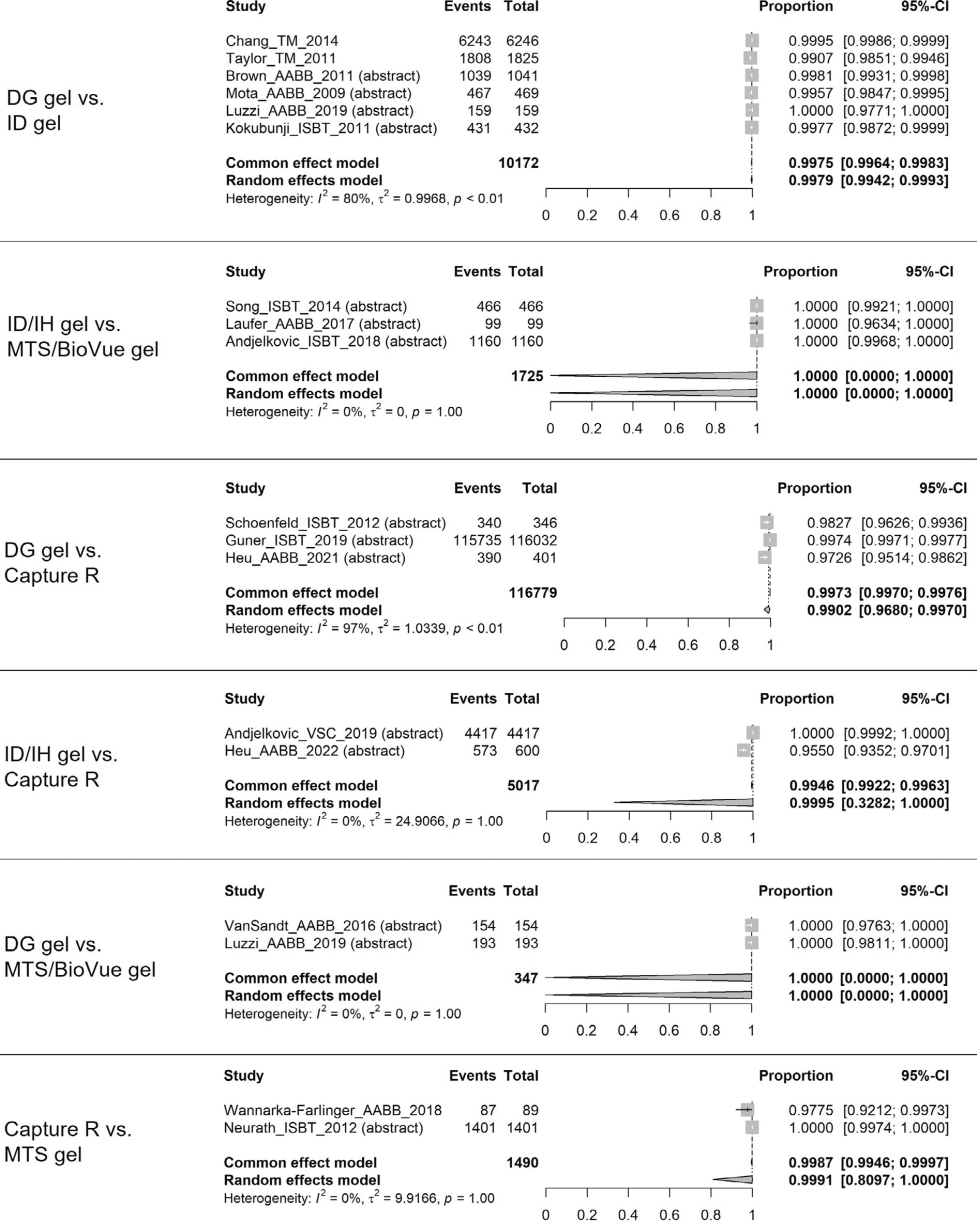
Concordance of ABO/RhD typing. *Source*: The event is the number of samples that had concordant results; the total was the total number of samples tested. The common‐effect model shows the pooled concordance rate estimated using a fixed‐effects model; the random‐effects model shows the pooled concordance rate estimated using a random‐effects model. CI, confidence interval.

The concordance rates of ABO/RhD typing were meta‐analysed for six concordance pairs (i.e., DG gel vs. ID gel, IH/ID gel vs. MTS/BioVue gel, DG gel vs. Capture R, ID/IH gel vs. Capture R, DG gel vs. MTS/BioVue gel, and Capture R vs. MTS gel) as shown in Figure 2.^6^, ^13^, ^33^, ^34^, ^35^, ^36^, ^37^, ^38^, ^39^, ^40^, ^41^, ^42^, ^43^, ^44^, ^45^, ^46^, ^47^ The pooled random‐effect estimates for all six concordance pairs were greater than 99.00% (Supplementary material [Supplementary Table 14]).

The IH/ID gel versus MTS/BioVue gel and DG gel versus MTS/BioVue gel analyses had 100% concordance rates from all studies included, leading to inestimable 95% CIs. The heterogeneity (*I* ^2^) was high for DG gel versus ID gel and DG gel versus Capture R; however, this was due to close to 100% event rates in all studies rather than large variations among the studies.

#### 3.2.2 Concordance of antibody screening

**FIGURE 3:**
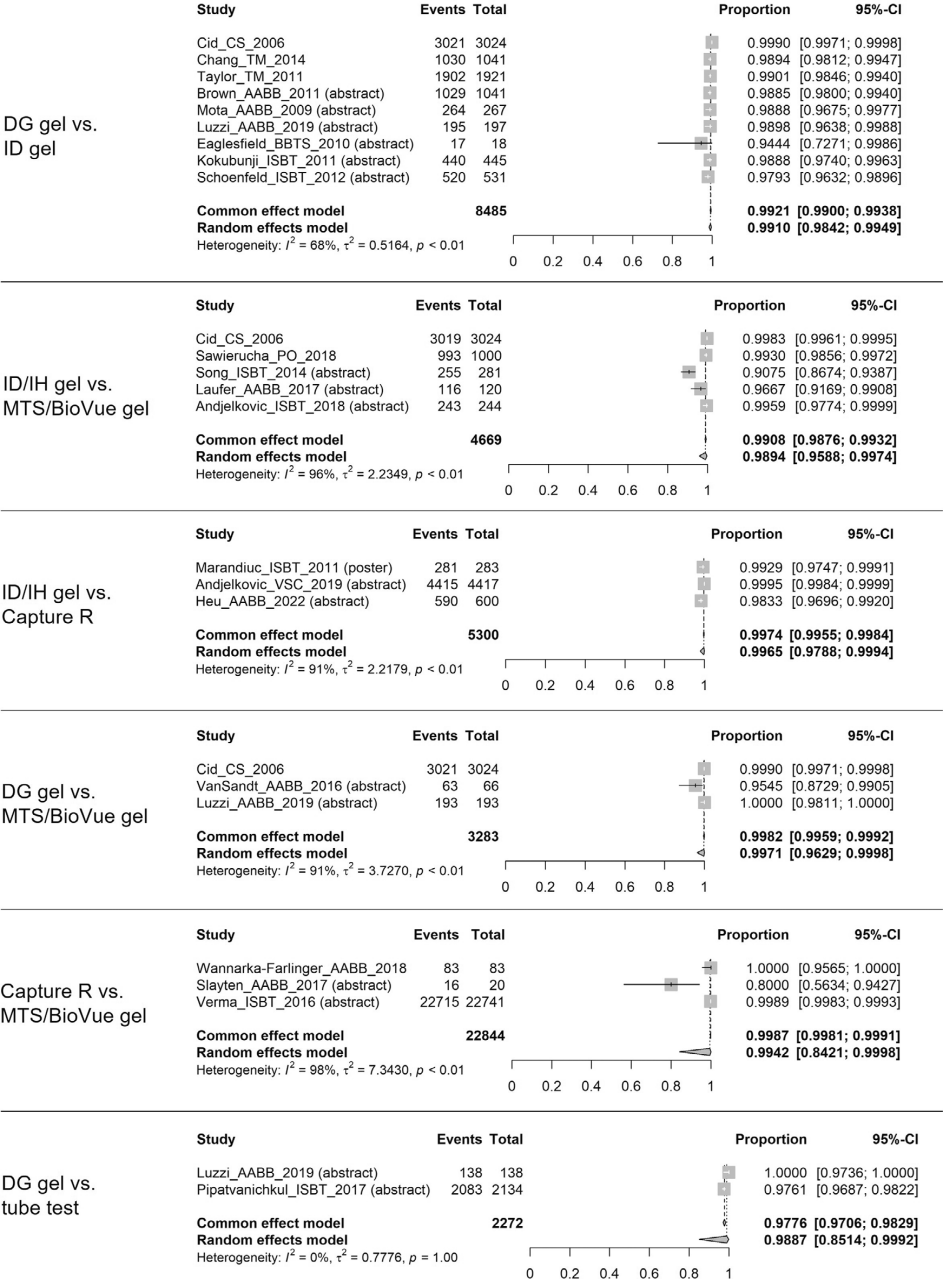
Concordance of antibody screening. *Source*: The event is the number of samples that had concordant results; the total was the total number of samples tested. The common‐effect model shows the pooled concordance rate estimated using the fixed‐effects model; the random‐effects model shows the pooled concordance rate estimated using the random‐effects model. CI, confidence interval.

The concordance rates of antibody screening were meta‐analysed for six concordance pairs (i.e., DG gel vs. ID gel, ID/IH gel vs. MTS/BioVue gel, ID/IH gel vs. Capture R, DG gel vs. MTS/BioVue gel, Capture R vs. MTS/BioVue gel, and DG gel vs. tube test), as shown in Figure 3.^6^, ^12^, ^13^, ^33^, ^34^, ^35^, ^36^, ^37^, ^38^, ^39^, ^40^, ^41^, ^42^, ^45^, ^47^, ^48^, ^49^, ^50^, ^51^, ^52^, ^53^ The pooled random‐effect estimates for all six concordance pairs were around 99.00% (Supplementary material [Supplementary Table 15]).

The heterogeneity (I^2^) was high in most analyses; however, this was mostly due to close to 100% event rates in all studies rather than large variations among the studies.

#### 3.2.3 Concordance of antibody identification

**FIGURE 4:**
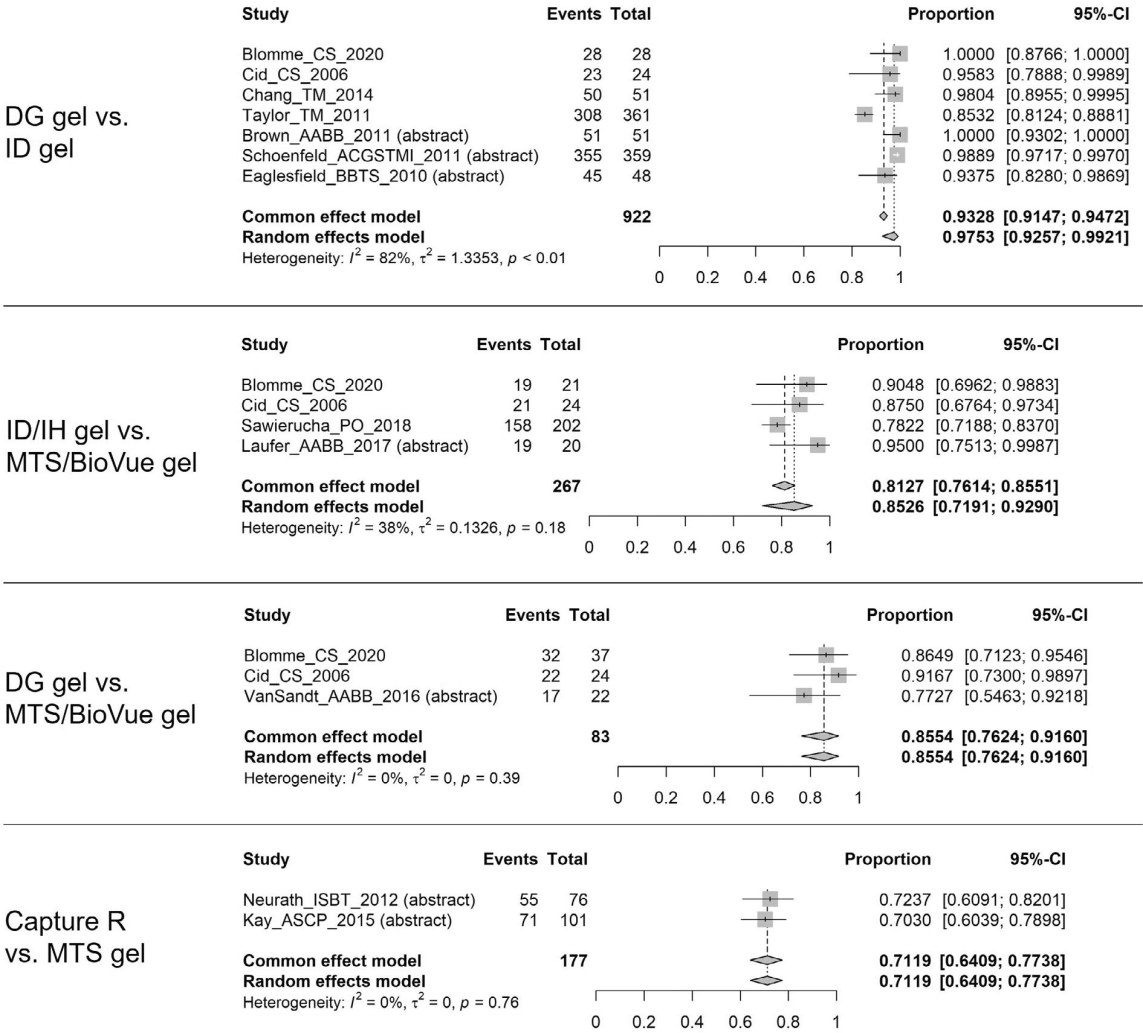
Concordance of antibody identification. *Source*: The event is the number of samples that had concordant results; the total was the total number of samples tested. The common‐effect model shows the pooled concordance rate estimated using a fixed‐effects model; the random‐effects model shows the pooled concordance rate estimated using a random‐effects model. CI, confidence interval.

The concordance rates of antibody identification were meta‐analysed for four concordance pairs (i.e., DG gel vs. ID gel, ID/IH gel vs. MTS/BioVue gel, DG gel vs. MTS/BioVue gel, and Capture R vs. MTS gel), as shown in Figure 4.^6^, ^8^, ^12^, ^13^, ^35^, ^36^, ^41^, ^44^, ^48^, ^53^, ^54^, ^55^ The pooled random‐effect estimates were 97.53% for DG gel vs. ID gel, 85.26% for ID/IH gel vs. MTS/BioVue gel, 85.54% for DG gel vs. MTS/BioVue gel, and 71.19% for Capture R vs. MTS gel (Supplementary material [Supplementary Table 16]).

High between‐study heterogeneity was observed in the analysis of DG gel vs. ID gel with an I^2^ of 81.82%.

#### 3.2.4 Sensitivity and specificity of antibody screening

**FIGURE 5:**
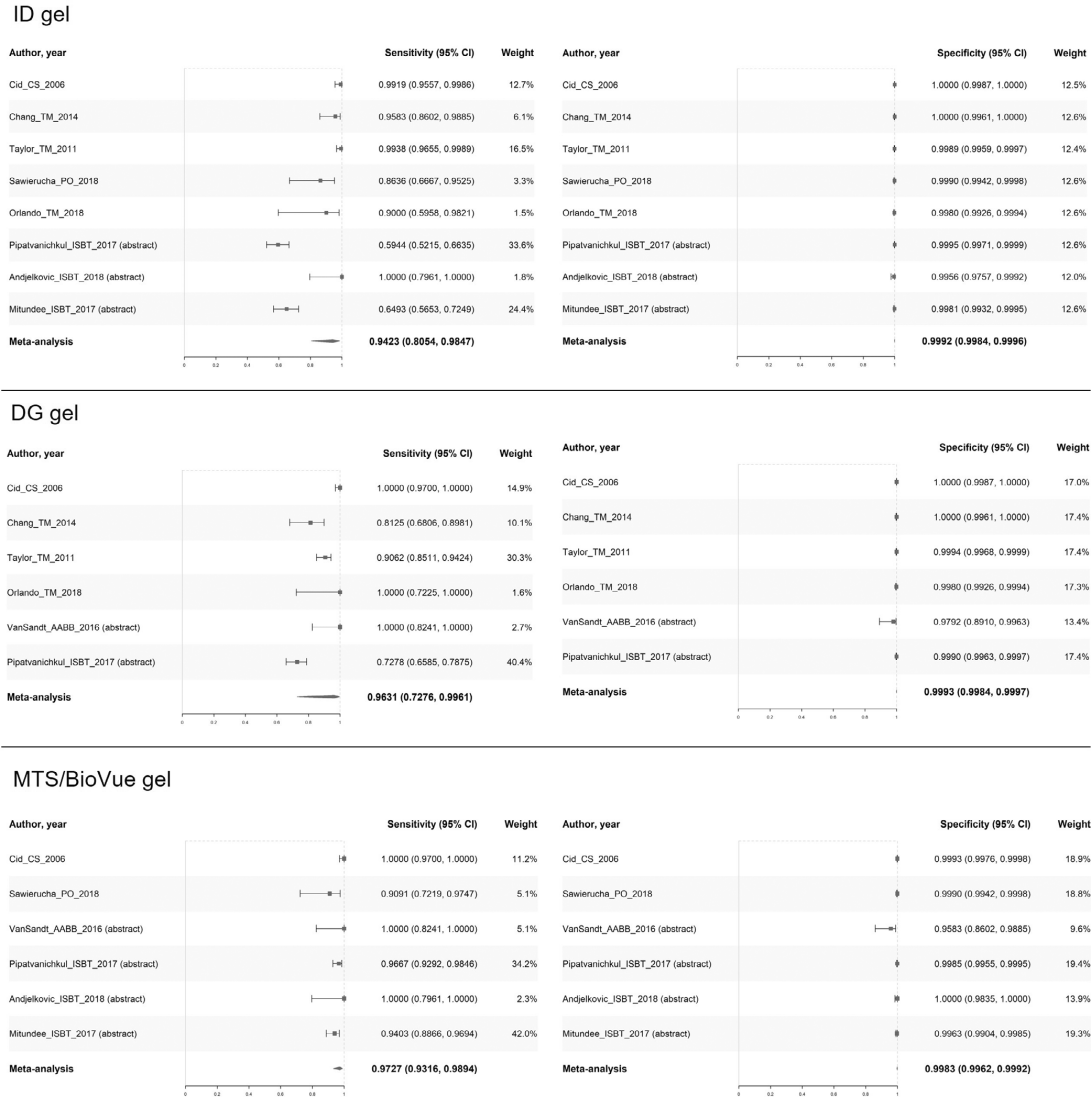
Sensitivity and specificity of antibody screening. CI, confidence interval.

The sensitivity and specificity of antibody screening were meta‐analysed for three index tests (i.e., ID gel, DG gel, and MTS/BioVue gel), as shown in Figure 5.^6^, ^12^, ^13^, ^33^, ^35^, ^48^, ^49^, ^56^, ^57^

Using the random‐effects bivariate binomial model to synthesise the sensitivity and specificity data, ID gel, DG gel, and MTS/BioVue gel had pooled sensitivities of 94.23%, 96.31%, and 97.27%, respectively. All three tests had close to 100% pooled specificity (Supplementary material [Supplementary Table 17]).

A description of the false positives and false negatives in the antibody screening is provided in Table 1.

**TABLE 1:** Ab screening summary.

References	Index test	Total sample	FP	FP Ab	FN	FN Ab
Cid_CS_2006^12^	Grifols	3124	0		0	
Cid_CS_2006^12^	Bio‐Rad	3124	0		1	1 anti‐JK
Cid_CS_2006^12^	Ortho	3124	2	1 panagglutinin 1 unspecified agglutination	0	
Chang_TM_2014^6^	Grifols	1041	0		9	9 weak prophylactic anti‐D
Chang_TM_2014^6^	Bio‐Rad	1041	0		2	1 anti‐e 1 anti‐JK
Taylor_TM_2011^13^	Grifols	1921	1	1 DAT	15	1 sample allo‐anti‐e and auto‐anti‐c 1 LISS dependent panagglutination 2 anti‐K 11 anti‐D (3 weak alloimmune anti‐D and 8 residual prophylactic anti‐D)
Taylor_TM_2011^13^	Bio‐Rad	1921	2	No information	1	1 DAT
Sawierucha_PO_2018^48^	Bio‐Rad	1000	1	No Ab identified	3	1 Lua 1 K 1 WAA
Sawierucha_PO_2018^48^	Ortho	1000	1	No Ab identified	2	1 D + E 1 WAA
Orlando_TM_2018^56^	Grifols	986	2	Positive test was not confirmed at the subsequent step of antibody identification	0	‐
Orlando_TM_2018^56^	Bio‐Rad	986	2	Positive test was not confirmed at the subsequent step of antibody identification	1	Anti Kpa
Orlando_TM_2018^56^	Immucor	986	7	Positive test was not confirmed at the subsequent step of antibody identification	1	‐
VanSandt_AABB_2016 (abstract)^35^	Grifols	66	1	No identification of Ab	0	‐
VanSandt_AABB_2016 (abstract)^35^	Ortho	66	2	No identification of Ab	0	‐
Pipatvanichkul_ISBT_2017 (abstract)^49^	Grifols	2134	2	No information	49	No information
Pipatvanichkul_ISBT_2017 (abstract)^49^	Bio‐Rad	2134	1	No information	73	No information
Pipatvanichkul_ISBT_2017 (abstract)^49^	Ortho	2134	3	No information	6	No information
Andjelkovic_ISBT_2018 (abstract)^33^	Bio‐Rad	244	1	Non‐specific reasons	0	‐
Andjelkovic_ISBT_2018 (abstract)^33^	Ortho	244	0	‐	0	‐
Mitundee_ISBT_2017 (abstract)^57^	Bio‐Rad	1204	2	No information	47	No information
Mitundee_ISBT_2017 (abstract)^57^	Ortho	1204	4	No information	8	No information

Abbreviations: Ab, antibody; DAT, direct anti‐globin test; FN, false negative, FP, false positive; LISS, low‐ionic strength solution; WAA, warm‐reactive autoantibody.

## 4 DISCUSSION

This study provided comprehensive analyses of pretransfusion tests by different manufacturers, in terms of concordance for ABO/RhD typing, antibody screening and antibody identification, and sensitivity and specificity for antibody screening. This is the first study to synthesise the available evidence for the performance of different IH analysers for pretransfusion testing.

Among all concordance pairs analysed, ID/IH gel, DG gel, MTS/BioVue gel, and Capture R all had close to 100% pooled concordance with each other in ABO/RhD typing and antibody screening. Considering that ABO/RhD typing and antibody screening are key pretransfusion testing steps to prevent adverse transfusion reactions due to incompatible blood, all commercial tests by different companies are expected to identify the right blood types and existence of antibodies, and therefore, have good concordance.

Antibody identification had more varied results across studies and lower pooled concordance rates, with 97.53% for DG gel versus ID/IH gel, 85.26% for ID/IH gel versus MTS/BioVue gel, 85.54% for DG gel versus MTS/BioVue gel, and 71.19% for Capture R versus MTS/BioVue gel. According to experts in pretransfusion testing, compared with ABO/RhD typing and antibody screening, the performance of antibody identification is more likely to depend on the variable ability of IH analysers to identify specific antigens, the variation in reagent cells for Ab identification, the variable expertise of the laboratory technicians involved in the studies, or a combination of these factors.

Meta‐analysis of sensitivity and specificity was conducted for antibody screening, despite the limitation of lack of a common reference standard. ID/IH gel, DG gel, and MTS/BioVue gel had pooled sensitivities of 94.23%, 96.31%, and 97.27%, respectively, with a pooled specificity of close to 100% for all three tests. Since the meta‐analysis for each index test was conducted independently, the pooled sensitivity and specificity were not comparable across different index tests; therefore, a higher estimate of pooled sensitivity or specificity of one test does not necessarily suggest better performance over another test.

## 5 STRENGTHS AND LIMITATIONS

The identification of eligible studies followed a rigorous, systematic process; a comprehensive feasibility assessment was performed to select the appropriate meta‐analytical approaches for concordance and sensitivity/specificity, respectively. This is the first SLR and meta‐analysis to assess different technologies.

This study, however, should be viewed in the context of the following limitations. A large proportion of included studies were only published as conference abstracts from peer‐reviewed journals and provided limited study details. No additional attempt (either by directly contacting the study authors or by performing follow‐up searches) was made by the authors of the current study to determine if any of the conference abstracts were reported as full‐text publications. Hand searches were performed only for conference abstracts published from 2021 to 2022 since abstracts older than two years were assumed to be indexed in Embase and, therefore, captured at the time of the database searches. Some analysers are only available in specific regions, which may impact the generalisability of the results. There was heterogeneity in how the results of diagnostic testing were reported, and assumptions and/or calculations were made for many data elements at the data extraction stage, which may limit the comparability among studies. There was a lack of eligible studies reporting the same IH tests of interest, necessitating the IH tests to be grouped by manufacturer for meta‐analysis. However, differences among products by the same manufacturer were expected to be small (due to the use of the same technology) as validated by experts in pretransfusion testing.

In concordance analysis, many concordance pairs of IH tests were only reported in a limited number of studies (≤3), and concordance pairs reported in only one study were not eligible for meta‐analysis. Concordance rates were pooled across studies rather than using Kappa statistics. Although the Kappa statistics approach considers both concordant positive and concordant negative and could be a better measure of this outcome, it was not analysed due to the scarcity of Kappa data.

In sensitivity and specificity analysis, ABO/RhD typing was not meta‐analysed due to the scarcity of data. Antibody identification was not meta‐analysable because the majority of the studies focused on the samples that were already antibody screening positive, leading to only sensitivity data being available, while both sensitivity and specificity were required for meta‐analysis.

Reference standards in the sensitivity and specificity analysis of antibody screening varied across the studies. The reference standard used in each study was assumed to show the true value, which limited the comparability between studies.

The meta‐analytical approaches used in this study limited the concordance analysis to only two IH tests at a time, or the sensitivity/specificity analysis to only one index test at a time. Therefore, comparison or ranking among multiple IH tests was not feasible with the current analytical setting, which limits the ability to use the results herein to inform decision‐making on choosing the best product. Although network meta‐analysis (NMA) could be used to form networks of all available tests, NMA for concordance rates was not feasible due to the lack of relative effect measures or arm‐level data from concordance. The NMA method for sensitivity and specificity is still in development by researchers, and there are currently no guidelines or validated statistical methods. Additionally, the common existence of 100% sensitivity and specificity was likely to lead to model convergence issues.

Bivariate binomial models were used to meta‐analyse sensitivity and specificity together considering their correlation, which was different from traditional meta‐analysis. Although this was the approach recommended by the Cochrane Group,^28^ there is a need for official guidelines from health technology assessment agencies.

## 6 CONCLUSIONS

This study provided a comprehensive assessment of concordance and diagnostic accuracy for four pretransfusion IH tests: DG gel, MTS/BioVue gel, ID/IH gel, and Capture R. All four tests had good concordance in ABO/RhD typing and antibody screening, and lower pooled concordance rates for antibody identification. For antibody screening, DG gel, MTS/BioVue gel, and ID/IH gel demonstrated an estimated 95% pooled sensitivity and close to 100% specificity.

Further research that directly compares multiple IH tests in the same study setting is warranted. Although the tube test is generally agreed as the gold standard, the reference used in the comparative studies was variable, underscoring the need for more consensus in the field on reference standards and in consistent reporting of outcomes.

## AUTHOR CONTRIBUTIONS

Christopher Elliott and Stephanie Kelham contributed to the critical review of the research and to the interpretation of the data.

Liu Zhang, Stacy Grieve, Tommy Lan, and Hoora Moradian contributed to the design of the study, data extraction and analysis of data, and to the interpretation of the data. Cristina Coll‐Ortega and David Gómez‐Ulloa contributed to the concept and design of the study, data extraction, and to the interpretation of the data.

## FUNDING INFORMATION

This study was funded by Grifols.

## CONFLICT OF INTEREST STATEMENT

CCO and DGU are full‐time employees of Grifols and have no other conflict of interest.

## PATIENT CONSENT

Patient consent statement is not applicable.

## Supporting information


**Data S1.** Supporting information.

**Data S2.** Supporting information.

## Data Availability

The data that support the findings of this study are available from the corresponding author upon reasonable request.
