# Supplementary material for: Systematic literature review and meta‐analysis of concordance and accuracy of pretransfusion immunohematology routine tests
Source: Transfus Med. 2025 Aug 25;35(5):445–57. doi: 10.1111/tme.70010 (PMC12499635; doi:10.1111/tme.70010)
Supplement: Supplementary file 1 — Data S1. Supporting information. [file TME-35-445-s002.docx]

**Title:** Systematic literature review and meta-analysis of concordance and accuracy of pretransfusion immunohematology routine tests

# **Supplementary Material**

**Supplementary Figure 1. Pretransfusion testing process**

Supplementary Table 1. Literature sources

| Source | Database |
| --- | --- |
| Electronic databases (via Ovid) | - MEDLINE and MEDLINE In-process - Embase and Embase In-process - EBM Reviews: Cochrane Central Register of Controlled Trials - EBM Reviews: Cochrane Database of Systematic Reviews |
| Conferences | - All conferences indexed via Embase - Hand search of the proceedings of the last editions available until the day of search execution (2021–2022) of: - International Society of Blood Transfusion - Association for the Advancement of Blood & Biotherapies - British Blood Transfusion Society - American Association of Clinical Chemistry |
| Other sources | Hand-searching the bibliography of relevant SLRs/meta-analyses identified by the database searches |

Abbreviations: EBM, Evidence-Based Medicine; SLR, systematic literature review

Supplementary Table 2. Search strategies (search date: April 19, 2023)

| **#** | **Search terms** | **Hits** |
| --- | --- | --- |
| 1 | exp blood group typing/ or exp blood group system/ or exp "Blood Grouping and Crossmatching"/ or exp Blood Group Antigens/ or (blood type* or blood group determination or blood group identification or blood group serology or blood grouping or crossmatching or blood typing or serogrouping or blood crossmatch$).ti,ab. | 93,821 |
| 2 | exp blood group ABO system/ or exp ABO Blood-Group System/ or ("abh blood group" or "abh human blood group" or "ABO blood group" or "ABO" or "abo blood group system" or "abo blood system" or "abo human blood group" or "abo human blood group system" or "abo system" or "blood group abo" or "serum system abo").ti,ab. | 47,132 |
| 3 | exp blood group rhesus system/ or exp Rh-Hr Blood-Group System/ or ("blood group cde" or "cde" or "blood group r" or blood group rhesus or "blood group rhesus gene cde" or "rhesus blood group" or "RhD" or Rh phenotyp? or "blood group rh system" or "blood group rhesus factor" or "blood group system rh" or "blood group system rhesus" or "rh blood group system" or "rh d system" or "rh d" or "rh system" or "rhesus blood group system" or "rhesus group" or "rhesus system").ti,ab. | 31,494 |
| 4 | exp erythrocyte antibody/ or exp erythrocyte antigen/ or (erythrocyte antibod* or "erythrocyte antiserum" or red blood cell antibod* or "red blood cell antiserum" or red cell antibod$4 or erthyrocyte antigen or "erythrocyte antigens" or "red blood cell antigen" or "red blood cell antigens" or red cell antigen$2).mp. | 9,065 |
| 5 | exp rhesus D antibody/ | 4,475 |
| 6 | exp agglutination test/ or exp Agglutination Tests/ or exp Coombs test/ or (antiglobulin test* or column agglutination technology or anti-globulin consumption test*).ti,ab. or ((anti?globulin or anti?immunoglobulin) adj2 test*).ti,ab. or (coomb$ adj2 (serum or reaction or test)).ti,ab. | 63,451 |
| 7 | exp antibody screening/ or (antibody screen* or antibody identification or antibody panel or AHG test* or AHG crossmatch*).ti,ab. | 14,199 |
| 8 | 1 or 2 or 3 or 4 or 5 or 6 or 7 | 193,619 |
| 9 | immunoh?ematology testing.mp. | 82 |
| 10 | exp immunohematology analyzer/ or (immunoh?ematology or blood bank analy#er* or immunoh?ematology analy#er* or automated blood bank analy#er* or semi-automated blood bank analy#er* or automated immunoh?ematology or semi-automated immunoh?ematology).mp. | 2,543 |
| 11 | (DG gel or DG-Gel or "gel card").mp. | 528 |
| 12 | ("ID-MTS" or "MTS-Gel" or "gel test" or "card test" or "MTS buffered Gel" or (MTS adj2 (gel or anti-IgG))).mp. | 1,962 |
| 13 | (IH-Gel or IH-500 or IH-1000 or IH-Card).mp. | 99 |
| 14 | (ID-gel or Bio-Rad gel card$).mp. | 88 |
| 15 | (biovue or "ortho sera" or Ortho BioVue or ortho vision analy#er or "ortho vision").mp. | 244 |
| 16 | (Capture-R or "capture R" or Ready-ID or column agglutination technology or liquid phase microplate).mp. | 433 |
| 17 | Erytra.mp. | 67 |
| 18 | 9 or 10 or 11 or 12 or 13 or 14 or 15 or 16 or 17 | 5,435 |
| 19 | 8 and 18 | 2,993 |
| 20 | (addresses or bibliography or biography or case report or comment or congresses or consensus development conference or duplicate publication or editorial or guideline or in vitro or interview or lectures or letter or monograph or news or "newspaper article" or practice guideline or "review literature" or "review of reported cases" or review, academic or review, multicase or review, tutorial or twin study).pt. | 4,727,994 |
| 21 | (animals/ not (humans/ and animals/)) or (animal/ not (human/ and animal/)) | 6,299,506 |
| 22 | case report/ or case reports/ | 5,220,452 |
| 23 | 20 or 21 or 22 | 15,608,184 |
| 24 | 19 not 23 | 2,470 |
| 25 | limit 24 to english language | 2,234 |
| 26 | limit 25 to yr="2006 -Current" | 1,848 |
| 27 | remove duplicates from 26 | 1,513 |

Supplementary Table 3. Inclusion/exclusion criteria for systematic literature review

| Domain | Inclusion criteria | Exclusion criteria |
| --- | --- | --- |
| Population | Human blood samples undergoing testing | Non-human studies |
| Intervention/ Comparator | Must compare at least two technologies (index and reference test must be clearly identified) for ABO/RhD typing, antibody screen, or antibody Identification.  North America:   - Grifols DG gel - Ortho MTS gel - Bio-Rad IH gel - Immucor Capture R - Immucor liquid phase microplate   Rest of the world:   - Grifols DG gel - Ortho BioVue gel - Bio-Rad ID gel - Immucor Capture R - Immucor liquid phase microplate | Not comparing at least two technologies (Studies comparing one automated immunohematology test to a manual reference standard were excluded.) |
| Outcomes | For the tests (ABO/RhD typing, antibody screen, antibody Identification):   - Sensitivity - Specificity - True positive/negative - False positive/negative - Positive/negative predictive value - Analysis of discordant results - Concordance/discordance results - Accuracy - Test turnaround time/hands-on time/maintenance time | Studies not reporting on the accuracy of immunohematology tests |
| Study design | Any comparative study | - Systematic literature reviews, and (network) meta-analyses* - Non-systematic reviews - Case series and reports, commentaries, editorials, and letters |
| Time period | - 2006 to April 2023 (full-text publications and indexed abstracts)^ - 2021 to 2022 (hand-search of conference abstracts) |  |

* The bibliographies of relevant systematic literature reviews, (network) meta-analyses and indirect treatment comparisons were cross-checked to ensure that no relevant articles were missed. However, these study designs were otherwise not included in the review to avoid double counting

^ Time-limit applied to correspond with the introduction of automated immunohematology testing

Supplementary Table 4. Technologies and analyzers reported in studies

| Company | Technology | Technology detail | Technology name reported in the study | Analyzer/automation system |
| --- | --- | --- | --- | --- |
| Grifols | DG gel | 8-column gel card using CAT | - DG gel - Identisera Diana (P) - Serascan Diana-4 (Micro-CAT) - IgG coombs gel card - WaDiana gel CAT - Gel Coombs cards (Gel/IAT) - CAT/CAT panel - Gel microfiltration technique | - Erytra - Erytra Eflexis - WADiana |
| Ortho | MTS gel (North America) BioVue gel (rest of the world) | ID-Micro Typing System (ID-MTS)-based CAT  BioVue system CAT | - MTS gel/ ID-MTS/ ID-MTS gel cards/ ID-MT-gel - BioVue/ BioVue® System cassettes - RESOLVE ® panel C poly cassettes - IgG gel cards - IgG coombs gel card - Glass bead-based CAT - Routine CAT - Gel panels - Gel technique | - ORTHO VISION - ORTHO VISION Max - AutoVue Innova - ProVue - Mitis-2 - Tecan MEGAFlex ID - Manual |
| Bio-Rad (including DiaMed) | IH gel (North America) ID gel (rest of the world) | CAT | - ID card/ Dia-Med-ID/ ID-system card - ID-DiaCell I-II/ ID-DiaCell I-III - ID-DiaPanel - ID-system CAT - ID-cards LISS/Coombs - IH gel - DiaMed gel centrifugation cards - LISS Coombs gel - Indirect Coomb's test on gel cards - CAT/CAT panel - Gel card/ Gel test | - Tango Optimo - IH-1000 - IH-500 - Techno Twin Station - Gel Station Classic - Manual - DiaMed ID-Micro Typing System (manual) |
| Immucor | Capture R | SPRCA; microplate hemagglutination technique | - Capture-R/ Capture-R Ready Screen/Capture - Capture-R Select Plate - Read-ID, Extend I, and Extend II (solid phase automated panel) - Ready ID panels (SPRCA) - Microplate hemagglutination technique - Microplate | - Galileo Echo/Echo - Galileo Neo/Neo - Echo Lumena (Echo 2.0) - Neo Iris - Manual |

Abbreviations: CAT, column agglutination technology; IgG, immunoglobin G; SPRCA, solid-phase red cell agglutination assay

Supplementary Table 5. Studies excluded due to additional selection criteria

| **Citation** | **Reason for not being included in the meta-analysis** |
| --- | --- |
| Haywood JR, et al. Determination of optimal method for antibody identification in a reference laboratory. Immunohematology. 2011;27(4):146-50. | Mixed technologies (≥2 as one comparator used in concordance) |
| Roback JD, et al. A multicenter study on the performance of a fully automated, walk-away high-throughput analyzer for pretransfusion testing in the US population. Transfusion. 2015;55(6 Pt 2):1522-8. | Mixed technologies (≥2 as one comparator used in concordance) |
| [Walton](https://doi.org/10.1111/j.1537-2995.2009.02366.x) L, et al. Three way manual technology comparison to support the purchase of an automated blood bank analyzer. Transfusion. Conference: AABB Annual Meeting and TXPO. New Orleans, LA United States. Conference Publication. 49(SUPPL. 3) (pp 145A-146A), 2009. | Mixed technologies (≥2 as one comparator used in concordance) |
| Slayten JK, et al. A comparison of automated platform testing of prenatal samples to standard PEG-AHG. Transfusion. Conference: AABB Annual Meeting and CTTXPO 2012. Boston, MA United States. Conference Publication. 52(SUPPL. 3) (pp 148A-149A), 2012. | No specificity data reported |
| Daniel N, et al. A comparison of column agglutination and solid phase red cell adherence technologies for red cell antibody detection. | No specificity data reported |
| Raos MR, et al. Vox Sanguinis. Conference: 31st Regional ISBT Congress, ISBT In Focus. Virtual. 116(SUPPL 1) (pp 23), 2021. | Comparator not clearly defined/not of interest |
| Milbradt-Pohan S, et al. Assessment of the bio-RAD IH system for blood grouping. Transfusion. Conference: AABB Annual Meeting 2014. Philadelphia, PA United States. Conference Publication. 54(SUPPL. 2) (pp 164A-165A), 2014. | ABO and RhD typing results reported separately |
| Aravechia M, et al. Comparison of the performance of two gel test systems for detection and identification of red cell clinically significant antibodies. Vox Sanguinis. Conference: 31st International Congress of the International Society of Blood Transfusion in Joint Cooperation with the 43rd Congress of the DGTI. Berlin Germany. Conference Publication. 99(SUPPL. 1) (pp 360), 2010. | Comparator not clearly defined/not of interest |
| Jung BK, et al. Comparison of three automated blood typing systems for ABO/D typing in ABO subgroup. Vox Sanguinis. Conference: 34th International Congress of the International Society of Blood Transfusion. Dubai United Arab Emirates. 111(Supplement 1) (pp 203), 2016. | Comparator not clearly defined/not of interest |
| Almgren J, et al. Comparison of three fully automated systems for immunohematology with the focus on two important aspects of capacity-efficiency and stress. | No specificity data reported  Only efficiency and TAT reported (no outcome of interest) |
| Schrieber D, et al. Transfusion. Conference: AABB Annual Meeting 2014. Philadelphia, PA United States. Conference Publication. 54(SUPPL. 2) (pp 173A-174A), 2014. | Outcome of interest not reported  Only TAT reported (no outcome of interest) |
| Castella M, et al. Evaluation of erytra - A fully automated analyzer for pre-transfusion compatibility tests using 8-column gel agglutination cards. Transfusion. Conference: AABB Annual Meeting and CTTXPO 2011. San Diego, CA United States. Conference Publication. 51(SUPPL. 3) (pp 177A), 2011. | Mixed technologies (≥2 as one comparator used in concordance) |
| Casina TS, et al. Evaluation of the impact of advancements of new automation on future challenges. Transfusion. Conference: AABB Annual Meeting 2017. San Diego, CA United States. 57(Supplement 3) (pp 224A), 2017. | Outcome of interest not reported  Only TAT extracted (no outcome of interest) |
| Casina TS, et al., Evaluation of turnaround time as a measure of instrument capability to meet workload needs in the immunohematology lab. Vox Sanguinis. Conference: 35th International Congress of the International Society of Blood Transfusion, ISBT 2018. Toronto, ON Canada. 113(Supplement 1) (pp 90), 2018. | Outcome of interest not reported  Only TAT reported (no outcome of interest) |
| Person R, et al. Implementation of immucor NEO automated platform for routine use in a big donor testing laboratory. Transfusion. Conference: AABB Annual Meeting and CTTXPO 2013. Denver, CO United States. Conference Publication. 53(SUPPL. 2) (pp 178A-179A), 2013. | Mixed technologies (≥2 as one comparator used in concordance)  Mixed reference standard (more than two technologies compared for ABO/RhD Typing, and antibody screening) |
| Kokubunji A, et al. Improved ABO/RH(D) typing in japanese population using grifols DG gel system. Vox Sanguinis. Conference: 32nd International Congress of the International Society of Blood Transfusion in Joint Cooperation with the 10th Congress of AMMTAC. Cancun Mexico. Conference Publication. 103(SUPPL. 1) (pp 200), 2012. | Mixed technologies (≥2 as one comparator used in concordance)  No two-way comparison (more than two technologies compared for ABO/RhD Typing, and antibody screening) |
| Boland L, et al. Local evaluation of the processing time of two immunohematology analyzers: Ortho vision versus IH-1000. International Journal of Laboratory Hematology. Conference: 31st Annual Meeting of the International Society for Laboratory Hematology. Brussels Belgium. 40(Supplement 2) (pp 20), 2018. | Outcome of interest not reported |
| Arroyo J, et al. Performance of the automated immunohematology analyzer with glass beads column agglutination technology. Vox Sanguinis. Conference: 32nd International Congress of the International Society of Blood Transfusion in Joint Cooperation with the 10th Congress of AMMTAC. Cancun Mexico. Conference Publication. 103(SUPPL. 1) (pp 201), 2012. | Mixed technologies (≥2 as one comparator used in concordance) |

Supplementary Table 6. Overview of studies identified by the systematic literature review

| Citation | Region | Concordance rate | | | Kappa statistics | Sensitivity and specificity | |
| --- | --- | --- | --- | --- | --- | --- | --- |
|  |  | ABO/RhD typing | Antibody screening | Antibody identification |  | ABO/RhD typing | Antibody screening |
| Andjelkovic_ISBT_2018 (abstract)^33^ | Rest of world | ✓ | ✓ | - | ✓ | - | ✓ |
| Andjelkovic_VSC_2019 (abstract)^49^ | Rest of world | ✓ | ✓ | - | - | - | - |
| Blomme_CS_2020^8^ | Rest of world | - | - | ✓ | - | - | - |
| Brown_AABB_2011 (abstract)^40^ | Rest of world | ✓ | ✓ | ✓ | - | - | - |
| Chang_TM_2014^6^ | Rest of world | ✓ | ✓ | ✓ | ✓ | ✓ | ✓ |
| Cid_CS_2006^12^ | Rest of world | - | ✓ | ✓ | - | - | ✓ |
| Eaglesfield_BBTS_2010 (abstract)^55^ | Rest of world | - | ✓ | ✓ | - | - | - |
| Guner_ISBT_2019 (abstract)^47^ | Rest of world | ✓ | - | - | - | - | - |
| Heu_AABB_2021 (abstract)^50^ | North America | ✓ | ✓ | ✓ | - | - | - |
| Heu_AABB_2022 (abstract)^51^ | North America | ✓ | ✓ | ✓ | - | - | - |
| Kay_ASCP_2015 (abstract)^58^ | North America | - | - | ✓ | - | - | - |
| Kokubunji_ISBT_2011 (abstract)^44^ | Rest of world | ✓ | ✓ | - | - | - | - |
| Laufer_AABB_2017 (abstract)^45^ | North America | ✓ | ✓ | ✓ | - | - | - |
| Luzzi_AABB_2019 (abstract)^43^ | Rest of world | ✓ | ✓ | - | - | - | - |
| Marandiuc_ISBT_2011 (poster)^52^ | Rest of world | - | ✓ | - | - | - | - |
| Martinez_AABB_2022 (abstract)^56^ | North America | - | - | ✓ | - | - | - |
| Mitundee_ISBT_2017 (abstract)^39^ | Rest of world | - | - | - | - | - | ✓ |
| Mota_AABB_2009 (abstract)^41^ | Rest of world | ✓ | ✓ | - | - | - | - |
| Neurath_ISBT_2012 (abstract)^48^ | North America | ✓ | - | ✓ | - | - | - |
| Orlando_TM_2018^36^ | Rest of world | - | - | - | - | - | ✓ |
| Pipatvanichkul_ISBT_2017 (abstract)^38^ | Rest of world | - | ✓ | - | ✓ | - | ✓ |
| Sawierucha_PO_2018^35^ | Rest of world | - | ✓ | ✓ | - | - | ✓ |
| Schoenfeld_ACGSTMI_2011 (abstract)^57^ | Rest of world | - | - | ✓ | - | - | - |
| Schoenfeld_ISBT_2012 (abstract)^46^ | Rest of world | ✓ | ✓ | - | - | - | - |
| Slayten_AABB_2017 (abstract)^53^ | North America | - | ✓ | - | - | - | - |
| Song_ISBT_2014 (abstract)^34^ | Rest of world | ✓ | ✓ | - | ✓ | ✓ | - |
| Taylor_TM_2011^13^ | Rest of world | ✓ | ✓ | ✓ | ✓ | - | ✓ |
| VanSandt_AABB_2016 (abstract)^37^ | North America | ✓ | ✓ | ✓ | ✓ | - | ✓ |
| Verma_ISBT_2016 (abstract)^54^ | Rest of world | - | ✓ | - | ✓ | - | - |
| Wannarka-Farlinger_AABB_2018 (abstract)^42^ | North America | ✓ | ✓ | - | - | - | - |

Supplementary Table 7. Quality Assessment of Diagnostic Accuracy Studies_2 Risk-of-bias Quality Appraisal

| **Study** | **Location** | **IH test** | **Patient selection** | **Index tests** | **Reference standard** | **Flow and timing** |
| --- | --- | --- | --- | --- | --- | --- |
| Blomme_CS_2020^8^ | Belgium | ABO/Rh(D) | Low | Low | Not applicable | Low |
| Cid_CS_2006^12^ | Spain | Ab screening | Low | Low | Low | Low |
| Cid_CS_2006^12^ | Spain | Ab identification | Low | Low | Low | Low |
| Chang_TM_2014^6^ | UK | ABO/Rh(D) | Low | Low | Low | Low |
| Chang_TM_2014^6^ | UK | Ab screening | Low | Low | Low | Low |
| Chang_TM_2014^6^ | UK | Ab identification | Low | Low | Low | Low |
| Taylor_TM_2011^13^ | UK | ABO/Rh(D) | Low | Low | Unclear | Low |
| Taylor_TM_2011^13^ | UK | Ab screening | Low | Low | Low | Unclear |
| Taylor_TM_2011^13^ | UK | Ab identification | Low | Low | Unclear | Low |
| Sawierucha_PO_2018^35^ | Germany | Ab screening | Low | Low | Low | Unclear |
| Sawierucha_PO_2018^35^ | Germany | Ab identification | Low | Low | Not applicable | Low |
| Orlando_TM_2018^36^ | Italy | Ab screening | Low | Low | Low | Low |

Abbreviations: Ab, antibody; IH, immunohematology; UK, United Kingdom

Supplementary Table 8. Concordance rates for ABO/RhD typing reported in included studies

| **Citation** | **Test 1** | **Test 2** | **Sample (N)** | **Concordant (N)** | **Concordant rate** |
| --- | --- | --- | --- | --- | --- |
| Chang_TM_2014^6^ | DG gel | ID gel | 6,246 | 6,243 | 99.95% |
| Taylor_TM_2011^13^ | DG gel | ID gel | 1,825 | 1,808 | 99.07% |
| Brown_AABB_2011 (abstract)^40^ | DG gel | ID gel | 1,041 | 1,039 | 99.81% |
| Mota_AABB_2009 (abstract)^41^ | DG gel | ID gel | 469 | 467 | 99.57% |
| Wannarka-Farlinger_AABB_2018 (abstract)^42^ | Capture R | MTS gel | 89 | 87 | 97.75% |
| VanSandt_AABB_2016 (abstract)^37^ | DG gel | MTS gel | 154 | 154 | 100.00% |
| Luzzi_AABB_2019 (abstract)^43^ | DG gel | ID gel | 159 | 159 | 100.00% |
| Luzzi_AABB_2019 (abstract)^43^ | DG gel | BioVue gel | 193 | 193 | 100.00% |
| Luzzi_AABB_2019 (abstract)^43^ | DG gel | Tube test | 138 | 136 | 98.55% |
| Song_ISBT_2014 (abstract)^34^ | ID gel | BioVue gel | 466 | 466 | 100.00% |
| Kokubunji_ISBT_2011 (abstract)^44^ | DG gel | ID gel | 432 | 431 | 99.77% |
| Laufer_AABB_2017 (abstract)^45^ | IH gel | MTS gel | 99 | 99 | 100.00% |
| Schoenfeld_ISBT_2012 (abstract)^46^ | DG gel | Capture R | 346 | 340 | 98.27% |
| Guner_ISBT_2019 (abstract)^47^ | DG gel | Capture R | 116,032 | 115,735 | 99.74% |
| Andjelkovic_ISBT_2018 (abstract)^33^ | ID gel | BioVue gel | 1,160 | 1,160 | 100.00% |
| Neurath_ISBT_2012 (abstract)^48^ | Capture R | MTS gel | 1,401 | 1,401 | 100.00% |
| Andjelkovic_VSC_2019 (abstract)^49^ | ID gel | Capture R | 4,417 | 4,417 | 100.00% |
| Heu_AABB_2021 (abstract)^50^ | DG gel | Capture R | 401 | 390 | 97.26% |
| Heu_AABB_2022 (abstract)^51^ | IH gel | Capture R | 600 | 573 | 95.50% |

Supplementary Table 9. Concordance rates for antibody screening reported in included studies

| **Citation** | **Test 1** | **Test 2** | **Sample (N)** | **Concordant (N)** | **Concordant rate** |
| --- | --- | --- | --- | --- | --- |
| Cid_CS_2006^12^ | DG gel | ID gel | 3,024 | 3,021 | 99.90% |
| Cid_CS_2006^12^ | DG gel | BioVue gel | 3,024 | 3,021 | 99.90% |
| Cid_CS_2006^12^ | ID gel | BioVue gel | 3,024 | 3,019 | 99.83% |
| Chang_TM_2014^6^ | DG gel | ID gel | 1,041 | 1,030 | 98.94% |
| Taylor_TM_2011^13^ | DG gel | ID gel | 1,921 | 1,902 | 99.01% |
| Brown_AABB_2011 (abstract)^40^ | DG gel | ID gel | 1,041 | 1,029 | 98.85% |
| Sawierucha_PO_2018^35^ | ID gel | BioVue gel | 1,000 | 993 | 99.30% |
| Mota_AABB_2009 (abstract)^41^ | DG gel | ID gel | 267 | 264 | 98.88% |
| Marandiuc_ISBT_2011 (poster)^52^ | ID gel | Capture R | 283 | 281 | 99.29% |
| Wannarka-Farlinger_AABB_2018 (abstract)^42^ | Capture R | MTS gel | 83 | 83 | 100.00% |
| VanSandt_AABB_2016 (abstract)^37^ | DG gel | MTS gel | 66 | 63 | 95.45% |
| Slayten_AABB_2017 (abstract)^53^ | Capture R | MTS gel | 20 | 16 | 80.00% |
| Luzzi_AABB_2019 (abstract)^43^ | DG gel | ID gel | 197 | 195 | 98.98% |
| Luzzi_AABB_2019 (abstract)^43^ | DG gel | BioVue gel | 193 | 193 | 100.00% |
| Luzzi_AABB_2019 (abstract)^43^ | DG gel | Tube test | 138 | 138 | 100.00% |
| Verma_ISBT_2016 (abstract)^54^ | Capture R | BioVue gel | 22,741 | 22,715 | 99.89% |
| Song_ISBT_2014 (abstract)^34^ | ID gel | BioVue gel | 281 | 255 | 90.75% |
| Eaglesfield_BBTS_2010 (abstract)^55^ | DG gel | ID gel | 18 | 17 | 94.44% |
| Kokubunji_ISBT_2011 (abstract)^44^ | DG gel | ID gel | 445 | 440 | 98.88% |
| Laufer_AABB_2017 (abstract)^45^ | IH gel | MTS gel | 120 | 116 | 96.67% |
| Schoenfeld_ISBT_2012 (abstract)^46^ | DG gel | ID gel | 531 | 520 | 97.93% |
| Pipatvanichkul_ISBT_2017 (abstract)^38^ | DG gel | Tube test | 2,134 | 2,083 | 97.61% |
| Pipatvanichkul_ISBT_2017 (abstract)^38^ | ID gel | Tube test | 2,134 | 2,060 | 96.53% |
| Pipatvanichkul_ISBT_2017 (abstract)^38^ | BioVue gel | Tube test | 2,134 | 2,125 | 99.58% |
| Andjelkovic_ISBT_2018 (abstract)^33^ | ID gel | BioVue gel | 244 | 243 | 99.59% |
| Andjelkovic_VSC_2019 (abstract)^49^ | ID gel | Capture R | 4,417 | 4,415 | 99.95% |
| Heu_AABB_2021 (abstract)^50^ | DG gel | Capture R | 401 | 395 | 98.50% |
| Heu_AABB_2022 (abstract)^51^ | IH gel | Capture R | 600 | 590 | 98.33% |

Supplementary Table 10. Concordance rates for antibody identification reported in included studies

| **Citation** | **Test 1** | **Test 2** | **Sample (N)** | **Concordant (N)** | **Concordant rate** |
| --- | --- | --- | --- | --- | --- |
| Blomme_CS_2020^8^ | DG gel | ID gel | 28 | 28 | 100.00% |
| Blomme_CS_2020^8^ | DG gel | BioVue gel | 37 | 32 | 86.49% |
| Blomme_CS_2020^8^ | ID gel | BioVue gel | 21 | 19 | 90.48% |
| Cid_CS_2006^12^ | DG gel | ID gel | 24 | 23 | 95.83% |
| Cid_CS_2006^12^ | DG gel | BioVue gel | 24 | 22 | 91.67% |
| Cid_CS_2006^12^ | ID gel | BioVue gel | 24 | 21 | 87.50% |
| Chang_TM_2014^6^ | DG gel | ID gel | 51 | 50 | 98.04% |
| Taylor_TM_2011^13^ | DG gel | ID gel | 361 | 308 | 85.32% |
| Brown_AABB_2011 (abstract)^40^ | DG gel | ID gel | 51 | 51 | 100.00% |
| Sawierucha_PO_2018^35^ | ID gel | BioVue gel | 202 | 158 | 78.22% |
| Martinez_AABB_2022 (abstract)^56^ | DG gel | Tube test | 22 | 22 | 100.00% |
| Martinez_AABB_2022 (abstract)^56^ | MTS gel | Tube test | 22 | 22 | 100.00% |
| VanSandt_AABB_2016 (abstract)^37^ | DG gel | MTS gel | 22 | 17 | 77.27% |
| Schoenfeld_ACGSTMI_2011 (abstract)^57^ | DG gel | ID gel | 359 | 355 | 98.89% |
| Eaglesfield_BBTS_2010 (abstract)^55^ | DG gel | ID gel | 48 | 45 | 93.75% |
| Laufer_AABB_2017 (abstract)^45^ | IH gel | MTS gel | 20 | 19 | 95.00% |
| Neurath_ISBT_2012 (abstract)^48^ | Capture R | MTS gel | 76 | 55 | 72.37% |
| Kay_ASCP_2015 (abstract)^58^ | Capture R | MTS gel | 101 | 71 | 70.30% |
| Heu_AABB_2021 (abstract)^50^ | DG gel | Capture R | 50 | 49 | 98.00% |
| Heu_AABB_2022 (abstract)^51^ | IH gel | Capture R | 35 | 31 | 88.57% |

Supplementary Table 11. Sensitivity and specificity for ABO/RhD typing reported in studies

| **Citation** | **Index test** | **Total sample** | **True positive** | **False positive** | **True negative** | **False negative** | **Sensitivity** | **Specificity** |
| --- | --- | --- | --- | --- | --- | --- | --- | --- |
| Chang_TM_2014^6^ | DG gel | 6,246 | 2,917 | 0 | 3,326 | 3 | 99.90% | 100.00% |
| Chang_TM_2014^6^ | ID gel | 6,246 | 2,917 | 0 | 3,327 | 2 | 99.93% | 100.00% |
| Song_ISBT_2014 (abstract)^34^ | ID gel | 466 | 458 | 0 | 8 | 0 | 100.00% | 100.00% |
| Song_ISBT_2014 (abstract)^34^ | DG gel | 466 | 458 | 0 | 8 | 0 | 100.00% | 100.00% |

Supplementary Table 12. Sensitivity and specificity for antibody screening reported in studies

| **Citation** | **Index test** | **Total sample** | **True positive** | **False positive** | **True negative** | **False negative** | **Sensitivity** | **Specificity** |
| --- | --- | --- | --- | --- | --- | --- | --- | --- |
| Cid_CS_2006^12^ | DG gel | 3,124 | 124 | 0 | 3,000 | 0 | 100.00% | 100.00% |
| Cid_CS_2006^12^ | ID gel | 3,124 | 123 | 0 | 3,000 | 1 | 99.19% | 100.00% |
| Cid_CS_2006^12^ | BioVue gel | 3,124 | 124 | 2 | 2,998 | 0 | 100.00% | 99.93% |
| Chang_TM_2014^6^ | DG gel | 1,041 | 39 | 0 | 993 | 9 | 81.25% | 100.00% |
| Chang_TM_2014^6^ | ID gel | 1,041 | 46 | 0 | 993 | 2 | 95.83% | 100.00% |
| Taylor_TM_2011^13^ | DG gel | 1,921 | 145 | 1 | 1,760 | 15 | 90.63% | 99.94% |
| Taylor_TM_2011^13^ | ID gel | 1,921 | 159 | 2 | 1,759 | 1 | 99.38% | 99.89% |
| Sawierucha_PO_2018^35^ | ID gel | 1,000 | 19 | 1 | 977 | 3 | 86.36% | 99.90% |
| Sawierucha_PO_2018^35^ | BioVue gel | 1,000 | 20 | 1 | 977 | 2 | 90.91% | 99.90% |
| Orlando_TM_2018^36^ | DG gel | 986 | 10 | 2 | 974 | 0 | 100.00% | 99.80% |
| Orlando_TM_2018^36^ | ID gel | 986 | 9 | 2 | 974 | 1 | 90.00% | 99.80% |
| Orlando_TM_2018^36^ | Capture R | 986 | 9 | 7 | 969 | 1 | 90.00% | 99.28% |
| VanSandt_AABB_2016 (abstract)^37^ | DG gel | 66 | 18 | 1 | 47 | 0 | 100.00% | 97.92% |
| VanSandt_AABB_2016 (abstract)^37^ | MTS gel | 66 | 18 | 2 | 46 | 0 | 100.00% | 95.83% |
| Pipatvanichkul_ISBT_2017 (abstract)^38^ | DG gel | 2,134 | 131 | 2 | 1,952 | 49 | 72.78% | 99.90% |
| Pipatvanichkul_ISBT_2017 (abstract)^38^ | ID gel | 2,134 | 107 | 1 | 1,953 | 73 | 59.44% | 99.95% |
| Pipatvanichkul_ISBT_2017 (abstract)^38^ | BioVue gel | 2,134 | 174 | 3 | 1,951 | 6 | 96.67% | 99.85% |
| Andjelkovic_ISBT_2018 (abstract)^33^ | ID gel | 244 | 15 | 1 | 228 | 0 | 100.00% | 99.56% |
| Andjelkovic_ISBT_2018 (abstract)^33^ | BioVue gel | 244 | 15 | 0 | 229 | 0 | 100.00% | 100.00% |
| Mitundee_ISBT_2017 (abstract)^39^ | ID gel | 1,204 | 87 | 2 | 1,068 | 47 | 64.93% | 99.81% |
| Mitundee_ISBT_2017 (abstract)^39^ | BioVue gel | 1,204 | 126 | 4 | 1,066 | 8 | 94.03% | 99.63% |

Supplementary Table 13. Reference standards for sensitivity and specificity of antibody screening

| **Citation** | **Reference standard** |
| --- | --- |
| Cid_CS_2006^12^ | Microtube tests performed in parallel |
| Chang_TM_2014^6^ | If reactions gave results of opposite sign in each system (i.e., negative in one method and positive in the other), the result was considered a discrepancy. In such a case, a repetition in manual technique was carried out and the obtained result was taken as the true value. |
| Taylor_TM_2011^13^ | The reference method was DiaMed-ID® (DiaMed GMBH, Cressier, Switzerland) cards in all cases except in a small number of newborn tests in which reference method was the conventional tube technique. |
| Sawierucha_PO_2018^35^ | Antibody screening was done prospectively. When discrepant results were found (one instrument showed positive screening and the other negative), antibody identification was performed by both instruments. The test was classified as false negative when the antibody was not able to be identified by any analyzer, even when one of them showed positive screening results |
| Orlando_TM_2018^36^ | In case of discrepancy in the antibody screening, antibody identification was carried out, in order to confirm the antibody screening results. |
| VanSandt_AABB_2016 (abstract)^37^ | Not reported |
| Pipatvanichkul_ISBT_2017 (abstract)^38^ | Tube test |
| Andjelkovic_ISBT_2018 (abstract)^33^ | Not reported |
| Mitundee_ISBT_2017 (abstract)^39^ | Plasma samples with known results, so the true value was already identified before the testing |

Supplementary Table 14. Summary of pooled concordance of ABO/RhD typing

| **Comparison** | **Number of studies** | **Total concordance / Total sample** | **Pooled concordant rate**  **(95% CI) – random effects** | **Pooled concordant rate**  **(95% CI) – fixed effects** | **I^2^** |
| --- | --- | --- | --- | --- | --- |
| DG gel vs. ID gel | 6 | 10,147/10,172 | 99.79% (99.42%, 99.93%) | 99.75% (99.64%, 99.83%) | 80.47% |
| ID/IH gel vs. MTS/BioVue gel* | 3 | 1,725/1,725 | 100.00% | 100.00% | 0.00% |
| DG gel vs. Capture R | 3 | 116,465/116,779 | 99.02% (96.80%, 99.70%) | 99.73% (99.70%, 99.76%) | 97.47% |
| ID/IH gel vs. Capture R | 2 | 4,990/5,017 | 99.95% (32.82%, 100.00%) | 99.46% (99.22%, 99.63%) | 0.00% |
| DG gel vs. MTS/BioVue gel* | 2 | 347/347 | 100.00% | 100.00% | 0.00% |
| Capture R vs. MTS gel | 2 | 1,488/1,490 | 99.91% (80.97%, 100.00%) | 99.87% (99.46%, 99.97%) | 0.00% |

*The ID/IH gel vs. MTS/BioVue gel and DG gel vs. MTS/BioVue gel analyses had 100% concordance rate from all studies included, leading to inestimable 95% CIs.
Abbreviation: CI, confidence interval

Supplementary Table 15. Summary of pooled concordance of antibody screening

| **Comparison** | **Number of studies** | **Total concordance/ total sample** | **Pooled concordant rate**  **(95% CI) – random effects** | **Pooled concordant rate**  **(95% CI) – fixed effects** | **I^2^** |
| --- | --- | --- | --- | --- | --- |
| DG gel vs. ID gel | 9 | 8,418/8,485 | 99.10% (98.42%, 99.49%) | 99.21% (99.00%, 99.38%) | 67.79% |
| ID/IH gel vs. MTS/BioVue gel | 5 | 4,626/4,669 | 98.94% (95.88%, 99.74%) | 99.08% (98.76%, 99.32%) | 95.81% |
| ID/IH gel vs. Capture R | 3 | 5,286/5,300 | 99.65% (97.88%, 99.94%) | 99.74% (99.55%, 99.84%) | 90.87% |
| DG gel vs. MTS/BioVue gel | 3 | 3,277/3,283 | 99.71% (96.29%, 99.98%) | 99.82% (99.59%, 99.92%) | 90.88% |
| Capture R vs. MTS/BioVue gel | 3 | 22,814/22,844 | 99.42% (84.21%, 99.98%) | 99.87% (99.81%, 99.91%) | 97.58% |
| DG gel vs. tube test | 2 | 2,221/2,272 | 98.87% (85.14%, 99.92%) | 97.76% (97.06%, 98.29%) | 0.00% |

Abbreviation: CI, confidence interval

Supplementary Table 16. Summary of pooled concordance of antibody identification

| **Comparison** | **Number of studies** | **Total concordance/ total sample** | **Pooled concordant rate**  **(95% CI) – random effects** | **Pooled concordant rate**  **(95% CI) – fixed effects** | **I^2^** |
| --- | --- | --- | --- | --- | --- |
| DG gel vs. ID gel | 7 | 860/922 | 97.53% (92.57%, 99.21%) | 93.28% (91.47%, 94.72%) | 81.82% |
| ID/IH gel vs. MTS/BioVue gel | 4 | 217/267 | 85.26% (71.91%, 92.90%) | 81.27% (76.14%, 85.51%) | 38.36% |
| DG gel vs. MTS/BioVue gel | 3 | 71/83 | 85.54% (76.24%, 91.60%) | 85.54% (76.24%, 91.60%) | 0.00% |
| Capture R vs. MTS gel | 2 | 126/177 | 71.19% (64.09%, 77.38%) | 71.19% (64.09%, 77.38%) | 0.00% |

Abbreviation: CI, confidence interval

Supplementary Table 17. Summary of pooled sensitivity and specificity of antibody screening

| **Index test** | **Number of studies** | **Pooled sensitivity (95% CI)** | **Pooled specificity (95% CI)** |
| --- | --- | --- | --- |
| ID gel | 8 | 94.23% (80.54%, 98.47%) | 99.92% (99.84%, 99.96%) |
| DG gel | 6 | 96.31% (72.76%, 99.61%) | 99.93% (99.84%, 99.97%) |
| MTS/BioVue gel | 6 | 97.27% (93.16%, 98.94%) | 99.83% (99.62%, 99.92%) |

Abbreviation: CI, confidence interval
